# Personalized, Predictive, Participatory, Precision, and Preventive (P5) Medicine in Rotator Cuff Tears

**DOI:** 10.3390/jpm11040255

**Published:** 2021-04-01

**Authors:** Umile Giuseppe Longo, Arianna Carnevale, Carlo Massaroni, Daniela Lo Presti, Alessandra Berton, Vincenzo Candela, Emiliano Schena, Vincenzo Denaro

**Affiliations:** 1 Department of Orthopaedic and Trauma Surgery, Campus Bio-Medico University, Via Álvaro del Portillo, 00128 Rome, Italy; arianna.carnevale@unicampus.it (A.C.); a.berton@unicampus.it (A.B.); v.candela@unicampus.it (V.C.); denaro@unicampus.it (V.D.); 2 Unit of Measurements and Biomedical Instrumentation, Campus Bio-Medico University, Via Álvaro del Portillo, 00128 Rome, Italy; c.massaroni@unicampus.it (C.M.); d.lopresti@unicampus.it (D.L.P.); e.schena@unicampus.it (E.S.)

**Keywords:** personalized medicine, predictive medicine, participatory medicine, precision medicine, preventive medicine, orthopedics, rehabilitation, rotator cuff

## Abstract

Rotator cuff (RC) disease is a common musculoskeletal disorder of the shoulder entailing pain, with reduced functionality and quality of life. The main objective of this study was to present a perspective of the current scientific evidence about personalized, predictive, participatory, precision, and preventive approaches in the management of RC diseases. The personalized, predictive, participatory, precision and preventive (P5) medicine model is an interdisciplinary and multidisciplinary approach that will provide researchers and clinicians with a comprehensive patrimony of knowledge in the management of RC diseases. The ability to define genetic predispositions in conjunction with the evaluation of lifestyle and environmental factors may boost the tailoring of diagnosis and therapy in patients suffering from RC diseases.

## 1. Introduction

Rotator cuff (RC) disease is a common musculoskeletal disorder of the shoulder entailing pain, reduced functionality, and quality of life [1]. Management of RC diseases is an emerging topic in medicine and surgery that embraces several fields ranging from biology, genetics, biomechanics, economy, statistics, and engineering [2,3,4,5,6].

Traditionally, the approach adopted by medicine in the management of diseases has been based on prevention, diagnosis, and treatment [7]. Nowadays, the concept of P5 medicine is becoming a prominent model in the healthcare system [7,8,9].

The P5 medicine approach could provide revolutionary support to orthopaedic surgeons, researchers, and other healthcare professionals in the management of RC injuries, e.g., optimizing clinicians’ decision-making and the choice of the most tailored treatments. At the same time, in this new perspective, the P5 medicine approach could provide to the patients a greater awareness and independence in the management of their health.

RC disease still has many areas of uncertainty [10,11,12,13,14,15,16]. Several aspects related to the etiology of the disease and the optimization of the treatment are still to be optimized. In everyday clinical practice, surgery is offered to patients with a RC injury that is the same for each patient [17,18,19,20]. Generally, the same physiotherapy protocols and surgical techniques are applied to patients who are unique for their characteristics [21,22,23,24].

The main objective of this study was to provide a perspective of the current scientific evidence about personalized, predictive, participatory, precision, and preventive approaches in the management of RC diseases.

## 2. P5 Medicine

P5 medicine is the evolution of P4 medicine. This last implies a personalized, predictive, preventive, and participatory approach, aiming at determining the best treatments which will be the most suitable for each patient. The “fifth P” has referred to different aspects. The P5 medicine approach has been extended to a population level, i.e., taking into account heritability, which is distinctive for each population, and the public health policy [9,25]. Others proposed the psycho-cognitive aspect as an additional P in the P5 medicine model; namely, a patient is characterized not only by biological and genetic factors, but a person is also a unique individual for his/her attitudes, emotions, and cognitive functions [8,26,27,28,29]. In recent years, precision medicine has gained momentum in health management. What, though, is the difference between precision medicine and personalized medicine? The terms “precision” and “personalized” are often used interchangeably as synonyms, opening broad debates on underlying ethical and social issues [30,31,32,33]. Both approaches propel health management towards a patient-centered model of care [32,34]. Precision medicine applies “omics” science at the population level to identify subgroups of patients that may differ in genetics or biology regarding the development of possible diseases, or that may differ in their response to a particular therapy [35,36]. On the other hand, personalized medicine focuses the attention mainly on the individual—rather than on the general population—also considering contextual factors such as concomitant therapies, metabolism, environment, and lifestyle [35,37].

Compared to traditional medicine, P5 medicine is based on a proactive rather than reactive approach [37]. This subtle distinction implies a relevant meaning that is revolutionizing disease management. Indeed, despite providing interventions during the acute phase of an illness and “re-acting” with treatment and prevention strategies valid for the general population, P5 medicine focuses on a single patient according to a one-dimensional clinical approach [37]. The key aspect of this emerging approach regards the paradigm shift toward increasingly patient-centered medicine, moving beyond the “one size fits all” view (i.e., each treatment has similar effects on the whole population) [7,37].

Also, the confluence of bioinformatics, technological progress, and engineering science with wellness and clinical research is fostering the implementation of intervention programs created ad-hoc for the patient. In this scenario, P5 medicine entails the gathering of a large amount of data for each individual, ranging from medical and clinical history, environmental and social condition, to genomics and biological characteristics [30,37,38]. In Table 1, the definitions of each approach of the P5 medicine model are presented.

## 3. P5 Medicine in the Management of RC Diseases

An overview of the P5 medicine approach in the management of RC diseases is reported in Table 2 and detailed below.

### 3.1. Personalized Medicine

Personalized medicine is an aspect of the P5 medicine model that is also gaining momentum in the management of patients with RC diseases. A feature of personalized medicine is evaluating both the variability and unicity of patients in diagnosis and treatment. Research in the orthopedic field is moving towards a better understanding of the musculoskeletal apparatus, bones’ and muscles’ structure and role, applying computational models and innovative imaging techniques [41,52,53]. Telemedicine is a promising solution that aims to provide personalized and supervised therapy, breaking down the patient–therapist distance barrier [46]. Also, this approach of medicine allows for genetic information, comorbidity and biological and psychological aspects to develop more effective interventions for RC diseases [44,47,48].

### 3.2. Predictive Medicine

Predictive medicine employs systemic approaches founded on endogenous factors, such as genes and genetic variations [44]. It also considers individual aspects, such as environmental and lifestyle exposures. In patients with full-thickness RC tears, levels of pain, health education, and employment status are predictive factors of their emotional and psychological health [42]. Although predictive medicine is conventionally described as techniques and investigations based on genetics, emerging biomedical imaging techniques, machine learning methods, and subject-specific simulations may provide useful information for the definition of predictive models for diagnostic, therapeutic, and surgical scopes [41,43,50,51,52]. MRI analyses have been used in studies aiming to individualize plausible risk factors as predictors for the allocation to surgery of patients with partial-thickness RC tears, or to predict preoperatively retear risks after surgery [45,49].

### 3.3. Participatory Medicine

Participatory medicine supports and promotes the education of patients in the self-management of their health status. Following this approach, the goal is to intensify the engagement, motivation, and empowerment of the patients, exploiting a multi-centered collaboration that includes clinicians, therapists, or caregivers. The continuous advancements of technology (e.g., wearable sensors, virtual reality, machine learning techniques, and cloud database sharing) are fostering the inclusion of individuals in a collaborative network focused on a more patient-centered and participatory medicine [43,46]. Accurate classifications of movements in patients with RC tears have been proposed using inertial sensors [43]. Wearable systems are excellent candidates for supporting remote patient–clinician relationships and greater adherence to the prescribed home rehabilitation therapy [43,46].

### 3.4. Precision Medicine

Precision medicine is an evolving field trying to provide a categorization of diseases into subgroups based on genomic information. Musculoskeletal pain is a disabling condition common in patients suffering from RC tears. Precision interventions for shoulder pain have been proposed in a pre-clinical trial supporting the interaction between genetic and biopsychological factors [48]. Moreover, although specific genes (e.g., the estrogen-related receptor β (ESRRB) gene) and genetic variants (e.g., SAP30BP, SASH1) are intrinsic risk factors for RC tearing, other comorbidities may also influence RC disease [44,54].

### 3.5. Preventive Medicine

Preventive medicine can identify risk factors at both population and individual levels to contrast disease development and adverse outcomes with early interventions and treatments. Inflammatory biomarkers have been used to determine high-risk groups, to prevent shoulder pain by associating the molecular profile with psychological components [48]. Tailored preventive factors may be depicted using computational modeling and advanced imaging techniques to optimize diagnostic and surgical interventions in the management of RC disease [52,53]. Indeed, the early definition of the best treatment may prevent side effects or slow down the progression of the disease.

## 4. Discussion

This study delineates current scientific evidence in the management of RC diseases in the framework of P5 medicine. The P5 model encompasses personalized, predictive, participatory, precision, and preventive medicine, aiming to figure out the complexity of disease in all its aspects and to use the retrieved information for more patient-centered and individualized care.

To the best of our knowledge, this is the first study that tries to contextualize RC diseases in such an emerging topic as P5 medicine. Previous works have investigated P5 medicine in other research fields, such as oncology [25,26], forensic sciences [55], or autoimmune diseases [9]. Although the “fifth P” in the P5 medicine model has referred to psycho-cognitive features [27] or population-level perspectives [9,56], the common denominator is the same, namely, optimizing wellness for all individuals and their empowerment in the healthcare decision-making process, working closely with clinicians.

The P5 medicine approach seems to be very similar to the traditional medicine perspective. Our findings in RC diseases management confirm that the P5 medicine model’s ultimate goal is the same as that of traditional medicine; the substantial difference is that nowadays, researchers, clinicians, and patients can take advantage of sophisticated investigation techniques provided by emerging technological developments [4,32,34].

In recent decades, telemedicine and eHealth have fostered the application of technologies for promoting wellness and high quality of life [57,58]. An important step forward is that eHealth interventions may have a great potentiality to increase patient engagement [46,59]. eHealth interventions include emerging telecommunication technologies (e.g., smartphones, social media), cloud databases, wearable systems, and mobile technology (i.e., mHealth) [4,8,60]. These facilities could be useful not only to improve patients’ experience and satisfaction, but also for the educational scope to positively impact their lifestyle and care process, and increase adherence to clinicians’ recommendations [39,61].

P5 medicine, in the ongoing era of big data, aspires to delineate tailored prevention, diagnosis, and therapy for each individual [36,62]. Big data concern complex combinations of a huge amount of data deriving from multiple sources and regarding genetics, medical conditions, treatment typology, and demographic and environmental information [34]. Big data analytics and bioinformatics are supported by the engineering, biological, and biomedical sciences, and can be used to identify personalized therapies, as suggested by the P5 medicine approach [30]. Several studies investigated the association between RC diseases and genetic variations [6,63,64,65]. In the era of P5 medicine, biobanks aim to retrieve biological samples (e.g., blood, tissues, RNA, DNA) for therapeutic or research scopes [66].

RC disease is a multifactorial disease in which several components contribute to the disease process, like genetics, lifestyle, and structural features [1,6]. The management of RC tears could greatly benefit from a holistic approach tailored to the patient, optimized by the sophisticated technology available today, to obtain more reliable and efficient results [34].

At present, there is no clear scientific explanation that clarifies why re-rupture may occur after RC surgery in some patients, even with the implementation of the same surgical procedures, restrictions, and postoperative physiotherapy [67]. This unresolved issue justifies the need to develop more personalized medicine for patients with RC disorders [4]. Probably, genetic factors are involved, characterizing the patient’s predisposition [6,67,68]. Moreover, identifying those patients who have a very high probability of RC retear would help both the physician and patients by avoiding recommending surgery to patients with a high probability of failure [36]. In this way, direct and indirect costs could be avoided. On average, recovery after RC surgery can take between six months to one year [69,70,71,72].

Postoperative periods and potential further surgery can lead to serious inconvenience for the patient in daily life, work, interpersonal relationships, and the family environment. Indeed, not all patients with RC tears need surgery [73]. There are many patients with an asymptomatic RC injury, and progression to a pseudo-paralytic shoulder occurs only in a small percentage of cases. Furthermore, there is no correlation between the structural integrity of the RC tendons, shoulder functionality, patient satisfaction, and the ability to carry out the activities of daily living independently [74]. Therefore, applying the principle of predictive medicine to identify patients who have a better chance of recovery with alternative treatments rather than surgery would be of great social value [36].

Furthermore, in the postoperative period, the motivation to perform physiotherapy can be very different. For this reason, telemedicine, remote monitoring of shoulder movements, and gamification could play an essential role in participatory medicine. It could result in increasing patients’ empowerment and engagement, and better outcomes [4,60].

Precision medicine in RC disease could lead to choosing the most suitable treatment for each patient, based on the characteristics of the tendon, fatty degeneration, genetic profile, probability to develop shoulder stiffness, biomarkers, and genomics.

Finally, preventive medicine may reduce the number of patients affected by RC disease. Preventive measures could include changing some environmental and behavioral features. Often, people perform incorrect repetitive movements during sports or at work, leading to an increased risk of developing the disease.

P5 medicine approach in the management of RC tears could have many advantages, but some limitations need to be overcome. The multidisciplinary nature of P5 medicine requires close collaboration between experts in different fields (e.g., orthopedic, genetic, and engineering), and between physicians and patients. Consequently, P5 medicine entails collecting a large amount of heterogeneous data. The collection, analysis, and interpretation of these data could be challenging, as well as the associated costs. Although the implementation of the P5 medicine approach in common clinical practice is challenging, this is a worthwhile effort to make to improve the clinical outcomes of patients with RC tears.

## 5. Conclusions

**Figure 1:**
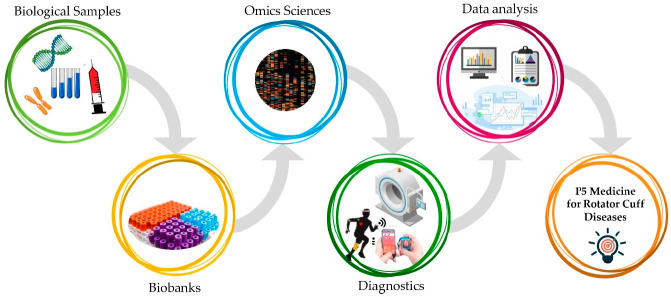
P5 medicine’s steps towards the optimal management of rotator cuff diseases.

The P5 medicine model is an interdisciplinary and multidisciplinary approach that will provide researchers and clinicians with a comprehensive patrimony of knowledge in the management of RC diseases (Figure 1). In this way, the ability to define the genetic predispositions in conjunction with the evaluation of lifestyle and environmental factors may boost the tailoring of the diagnosis and therapy in patients suffering from RC diseases. Future studies embracing clinical research, technology, and public health policy should intertwine more solidly to strengthen the clinician’s decision-making and to promote more individualized care for RC diseases.

## Figures and Tables

**Table 1:** Approaches of the personalized, predictive, participatory, precision and preventive (P5) medicine model.

Approach of P5 Medicine	Definition
Personalized	To identify tailored intervention based on the genetic profile of each individual, also considering other factors such as patients’ abilities, contexts, needs, social condition, lifestyle, family history, concomitant therapies, and psychological aspects [8,35,37].
Predictive	To exploit laboratory and genetic tests to predict the onset of a disease, and the deterioration or amelioration of a disease, by applying techniques and methods such as biomedical imaging instruments, artificial intelligence, and machine learning [7].
Participatory	To involve individuals in the management of their health status, strengthening the patients’ empowerment, autonomy, and engagement, and fostering the communication between multiple actors such as patients, doctors, and caregivers [39].
Precision	To classify patients with a disease into subgroups, considering their phenotypic findings, such as biomarkers or genomics [35].
Preventive	To define interventions for a specific pathology or disease before they occur, considering not only biological aspects but also environmental, social, and psychological aspects [40].

**Table 2:** Studies related to personalized, predictive, participatory, precision, and preventive approaches in the management of rotator cuff diseases. In light blue are highlighted the aspects of the P5 medicine approach emerging from each study.

First Author, Year of Publication	Personalized	Predictive	Participatory	Precision	Preventive	Main Purpose	Conclusions
Ameln, 2018 [41]						To use a musculoskeletal model, comprising a personalized scapulohumeral rhythm, to determine the stabilizing role of RC and selected superficial shoulder muscles as a function of humeral elevation and the plane of elevation.	The RC muscles provide greater compression than shear forces during tasks; the findings can be applied to understand how shear and compressive forces change in populations with abnormal shoulder motion.
Barlow, 2016 [42]						To establish factors most predictive of poor emotional health in patients with FTRCTs	Education level, employment status, pain levels, and patient perception of the percentage of shoulder normalcy were most predictive of emotional health in patients with FTRCTs.
Bavan, 2019 [43]						Demonstrating treatment fidelity to draw conclusions about the efficacy of rehabilitation interventions in both clinical and research settings	Classification models differentiated well patient exercise activity from non-specific movement by using inertial sensor data. Home rehabilitation could improve patient engagement.
Bonato, 2016 [44]						To investigate the association between TMD and RC disease and related genetic aspects.	TMD is a risk factor for RC disease. ESRRB haplotypes and low muscle activity are common biomechanical characteristics in subjects with both diseases.
Camurcu, 2019 [45]						To determine the predictive factors for allocation to surgery in patients older than 50 years with symptomatic chronic PTRCT.	Patients with fewer comorbidities and bursal-sided PTRCTs were significantly more likely to undergo surgery.
Carbonaro, 2018 [46]						To test a wearable technology-enabled platform for remote rehabilitation.	The proposed approach provides the patient and the therapist with relevant feedback on the quality of personalized rehabilitation.
Chiu, 2019 [47]						To develop a screening method for personalized best-fit PRFM to test as many tenocyte/PRFM interactions as possible.	The proposed platform could be used to screen personalized best-fit PRFM preparation protocols according to disease stage and severity.
George, 2017 [48]						To test the mechanisms and efficacy of personalized pain interventions matched to genetic and psychological characteristics.	This is a meaningful advance towards personalized or targeted treatments for musculoskeletal pain.
Jeong, 2018 [49]						To determine the risk factors related to retear after arthroscopic RC repair, to evaluate whether it is possible to predict the occurrence of retear preoperatively.	Predicting retear preoperatively may help surgeons determine the proper treatment and predict the postoperative prognosis.
Kang, 2017 [50]						To evaluate texture data of the torn supraspinatus tendon for prediction of the postoperative tendon state.	Texture analysis may be helpful to predict postoperative tendon state after RC repair.
Matcuk, 2019 [51]						To create predictive models to distinguish patients with RC tears from those without.	The proper combination of measurements on shoulder MRIs may be able to separate patients with RC tears from those without.
Zheng, 2019 [52]						To evaluate the effects of RC tear propagation on glenohumeral joint stability in a subject-specific finite element model of the shoulder complex.	This type of investigation can predict shoulder biomechanics, and can also be used to improve diagnostic and therapeutic strategies for clinicians.
Sakamoto, 2014 [53]						To evaluate the microstructure of the greater tuberosity of the humeral head in patients with RC tears, and to explore individual and regional variance of bone quality in vivo.	The proposed method can help to understand the individual and regional variance in bone quality and may lead to the creation of personalized surgical protocols.
Tashjian, 2016 [54]						To identify specific genes or genetic variants associated with rotator cuff tearing by a genome-wide association study.	Identification of potential genes or genetic variants associated with rotator cuff tearing may help in identifying individuals at risk of the development of rotator cuff tearing.

RC: rotator cuff, FTRCTs: full-thickness rotator cuff tears, TMD: temporo-mandibular disorders, ESRRB: estrogen-related receptor β, PTRCT: partial-thickness rotator cuff tear, PRFM: platelet-rich fibrin matrix.
